# Serum exosomal miRNA from endometriosis patients correlates with disease severity

**DOI:** 10.1007/s00404-021-06227-z

**Published:** 2021-09-20

**Authors:** Yahong Wu, Wen Yuan, Hui Ding, Xianqing Wu

**Affiliations:** Department of Obstetrics and Gynecology, The Second Xiangya Hospital of Central South University, 139 Middle Renming Road, Changsha, 430011 Hunan China

**Keywords:** Endometriosis, Exosomal miRNA, miRNA chip

## Abstract

**Purpose:**

Exosomes are vesicles secreted by cells that contain a wide variety of biomolecules, including proteins or nucleic acids. MicroRNAs (miRNAs), which are commonly found in exosomes, are known to play important roles in the pathophysiology of endometriosis.

**Methods:**

This study investigated the miRNA expression profile of serum exosomes from women with endometriosis in comparison with normal controls as well as the possible role of identified miRNAs in the pathogenesis of endometriosis. Exosomes with a diameter between 60 and 100 nm were identified by their expression of exosomal marker proteins CD9 and CD63.

**Results:**

Microarray miRNA expression profiling analysis revealed that 26 genes were significantly up-regulated and 19 genes were significantly down-regulated in serum exosomes from endometriosis patients compared with normal controls. These differentially expressed miRNAs were mainly enriched in the regulation of cellular development, metabolism, and involved in the regulation of the MAPK and PI3k-Akt pathways. qRT-PCR analysis verified the differential expression of three miRNAs, miR-26b-5p, miR-215-5p, and miR-6795-3p.

**Conclusion:**

Further analysis indicated that these differentially expressed miRNAs in serum exosomes may be involved in the pathogenesis of endometriosis and are related to the severity and certain symptoms of endometriosis.

**Supplementary Information:**

The online version contains supplementary material available at 10.1007/s00404-021-06227-z.

## Introduction

Exosomes are membrane vesicles with a diameter between 30 and 100 nm that are secreted by many cells within the body [1]. Exosomes have a lipid bimolecular layer structure and contain an abundance of information-carrying biomolecules, such as proteins, lipids, and nucleic acids. Accumulating evidence suggests that exosomes play important roles in antigen presentation, the immune response, protein and nucleic acid metabolism, intercellular communication, and the maintenance of homeostasis within the internal environment [2, 3]. While contained within exosomes, miRNAs generally remain safe from cleavage by RNA enzymes and can be released into specific recipient cells and tissues to mediate corresponding biological functions [4, 5].

Recently, studies have reported important functions of exosomes in tumors based on their regulation of a variety of biological behaviors, including the tumor microenvironment, vascular growth, metastasis, immune escape, and chemoresistance [6, 7]. Exosomes have been shown to be present in serum, plasma, ascites, cancer tissues, and cells [8, 9]. However, the roles of exosomal miRNAs in the field of gynecological diseases have not been fully investigated. Most current research has focused on the roles of exosomal miRNAs in ovarian cancer, endometrial cancer, and endometriosis. Exosomes derived from ovarian cancer cells were shown to promote intercellular signal transduction, which is mainly regulated by miRNAs within the exosomes. Taylor et al. [10] first reported a significant difference in the expression profile of miRNAs in the serum exosomes of ovarian cancer patients compared with normal controls and proposed the detection of specific miRNAs within blood exosomes as a novel method for the diagnosis of ovarian cancer. Abnormal miRNA expression in endometrial carcinoma was recently shown to mediate cancer stromal communication based on the action of metastasis-related miRNAs to promote the migration and invasion of endometrial carcinoma [11, 12]. Second-generation sequencing of the miRNA expression profiles of serum exosome from endometrial carcinoma patients revealed many differentially expressed miRNAs in the exosomes from these patients compared with the exosomes from normal controls. Among these miRNAs, miR-451a was identified as a potential molecular indicator for the early diagnosis of endometrial cancer and as a potential therapeutic target for endometrial cancer treatment [13].

Sun et al. [14] found that stromal cells in the eutopic endometrium may promote neuroangiogenesis through exosome release. Exosomes are a novel mode of communication between ESCs and macrophages, and the phagocytic ability of macrophages that absorbed exosomes from endometriotic tissue was decreased compared with that in the control group. These data indicate that exosomes from endometriotic tissue attenuate the phagocytic ability of macrophages and induce M2 macrophage polarization. Thus, exosomes from endometriotic tissue play a novel function in regulating macrophage activation and polarization, which could lead to the development of endometriosis [15]. Ectopic endometrial cells have been shown to secrete large numbers of exosomes in patients with endometriosis that contain many miRNAs related to the development of endometriosis. Harp et al. [16] reported significant differences in the miRNA contents of exosomes isolated from endometriosis patients compared with control individuals. For example, miRNA-21 expression in exosomes from endometriosis patients was 11 times higher than in the normal control group, and this miRNA is known to promote the growth, proliferation, and angiogenesis of ectopic stromal cells [17–20]. Exosome secretion has tissue and cell specificity, and thus, the analysis of exosomal miRNAs in patients with endometriosis can provide the basis for methods for early diagnostic screening, differential diagnosis, and treatment of the disease. The present study applied microarray chip technology to analyze the unique miRNA expression profile of serum exosomes from endometriosis patients relative to those from a control group.

## Material and methods

### Collection and disposal of specimens

The present study was approved by the Ethics Committee of Xiangya II Hospital of Central South University. A total of 42 patients with ovarian endometriosis who went to the gynecological hospital of Xiangya Second Hospital from January 2018 to December 2018 for laparoscopic surgery were enrolled. According to the American Society of Reproductive Medicine (rASRM) score, these cases included 12 stage I–II case and 30 stage III–IV cases. The control group consisted of 24 patients without endometriosis who sought treatment for a benign ovarian teratoma (*n* = 17) or a simple ovarian cyst (*n* = 7). For inclusion in the study, all patients had to meet the following criteria: (1) no use of hormone and hormone-like drugs within 3 months immediately before operation; (2) childbearing age (20–45 years) with a normal menstrual cycle; (3) absence of any other major systemic diseases, especially malignant tumors; and (4) consent to participate in the study after being informed of the process and purpose of intima collection. One case from each group was selected for exosome identification. A 10-ml blood sample was obtained in anticoagulation tubes from each patient. After centrifugation, the upper serum layer was collected and stored at − 80 °C for later analysis.

### Isolation and identification of serum exosomes

#### Exosome isolation

The patient’s serum samples were dissolved on ice and centrifuged at 10,0000 ×*g* for 30 min at 4 °C. The supernatant was then collected, and an exosome binding enhancer (Wako, Tokyo, Japan) was added according to the volume ratio of 1:500 prior to vortex mixing and setting aside. A balance solution consisting of 60 µL of magnetic beads and 500 µL of exosome trap was added, followed by vortex mixing. This solution was then left on a magnetic stand for 2 min, before removal of the supernatant and collection of the precipitant. Then 500 µL of exosome trap balance solution and 10 µL of biotin-labeled exosome trap was added for repeated vortex mixing and magnetic separation of the precipitant. Then 500 µL of exosome trap was added again for repeated vortex mixing and a further magnetic separation step. The sample was then subjected to vortex mixing for 3 h. After three washes in was buffer containing exosome binding enhancer (Wako) at a 1:500 volume ratio, exosomes were isolated from the supernatant.

#### Identification of exosomes

The exosome samples were dissolved in solution at 4 °C and then fixed with acid stationary solution, rinsed with phosphoric acid bleach solution three times, dehydrated in gradient ethanol solutions followed by acetone solutions, incubated with 100% acetone, and entrapped in solution at room temperature. The samples then polymerized in an oven, sliced, and washed for acetate staining. The samples were then incubated with lead acetate staining solution for 10 min, observed, and photographed by transmission electron microscopy.

#### Western blot validation of exosomes

The frozen exosome samples were dissolved, and 300 µL radioimmunoprecipitation assay (RIPA) buffer was added before repeated grinding of the sample in a homogenizer. After 30 min of protein cleavage, the samples were centrifuged at 12,000 ×*g* for 15 min at 4 °C. The total protein concentration was determined using a BCA protein quantification kit according to standard procedures. Then non-specific antigen was sealed by a nitrocellulose membrane and 5% skim milk powder. The membrane was washed and incubated with rabbit anti-human CD63 monoclonal antibody (1:1000, Abcam, Cambridge, MA, USA), rabbit anti-human CD9 monoclonal antibody (1:2000, Abcam), and rabbit anti-human GAPDH monoclonal antibody (1:4000, Abcam). The film was then washed and incubated with enhanced chemiluminescence (ECL) solution (Thermo) for 3 min.

### Chip analysis of miRNA expression profiles and RT-PCR verification

The total cell RNA was extracted by the TRIzol method after centrifugation at a low temperature. The obtained RNA was reversed to cDNA and stored at − 80 °C until use in further experiments. The extracted exosomal RNA was sent to Shanghai Kangcheng Biological Co. for miRNA chip analysis.

For RT-PCR analysis, the SYBR Green I dye method was used to prepare the primers. Template gene expression was used as the qPCR internal reference, and relative expression levels were calculated using the 2^–ΔΔCt^ method. The following primers (Sangon Biotech, Shanghai, China) for each gene were used:

(1) U6-forward: 5′-CTCGCTTCGGCAGCACA-3′;

U6-reverse: 5′-AACGCTTCACGAATTTGCGT-3′;

(2) hsa-miR-215-5p: GGCGGACCTATGAATTGACAGAC;

(3) hsa-miR-26b-5p: CCGGCCAAGTAATTCAGGATAGG;

(4) hsa-miR-6795-3p: ACCCCTCGTTTCTTCCCCCAG.

### Target gene prediction

#### Prediction data sources and strategies

We used two databases to predict the target genes of miRNAs: targetscan7.2 and mirdbV6. The miRNA–target interactions were then experimentally validated by the database mirTarbase7.0. (Targetscan7.2: the database Targetscan7.2 from http://www.targetscan.org/vert_71/.Mirdb: the database mirdbV6 is from http://mirdb.org/miRDB/).

#### Target gene ontology (GO) analysis and Kyoto encyclopedia of genes and genomes (KEGG) pathway analysis

The GO project provides a controlled vocabulary to describe gene and gene product attributes in any organism. The ontology covers three domains: biological process, cellular component, and molecular function. Pathway analysis is a functional analysis mapping genes to KEGG pathways. GO and KEGG pathway analyses were carried out to annotate the gene functions and pathways, respectively. To further explore the potential roles of significantly differentially expressed miRNAs in the pathogenesis of endometriosis, GO enrichment and KEGG pathway analyses were performed for the predicted target genes of significantly differentially expressed miRNAs. Two analyses denote the significance of the pathway correlated to the target genes, which means the selected miRNAs are likely to influence these pathways in the pathological process of endometriosis.

### Statistical analysis

SPSS 19.0 statistical software (SPSS, Inc., Chicago, IL, USA) was used for analysis, and all data are expressed as mean ± standard deviation (x ± s). Comparisons of the measurement data between the two groups were conducted using an independent samples *t* test. Comparisons of data for multiple samples were performed by univariate analysis of variance (one-way ANOVA). For pairwise comparisons between groups, the least significant difference (LSD) test was used for data with homogeneity of variance, and the Tamhane test was used for data with unequal variance. *P* < 0.05 was considered statistically significant.

## Results

After the isolation and identification of exosomes, 26 significantly up-regulated genes and 19 significantly down-regulated genes in the serum exosomes of endometriosis patients compared with controls were revealed through miRNA chip analysis. Then GO analysis and KEGG pathway analysis of the differentially expressed miRNA target genes revealed that they were related to certain functions and pathways. qRT-PCR analysis verified the differential expression of miR-26b-5p, miR-215-5p, and miR-6795-3p between endometriosis patients and controls, between cases of differing severity and according to the presence of dysmenorrhea or infertility.

### Verification of exosome isolation

**Fig. 1:**
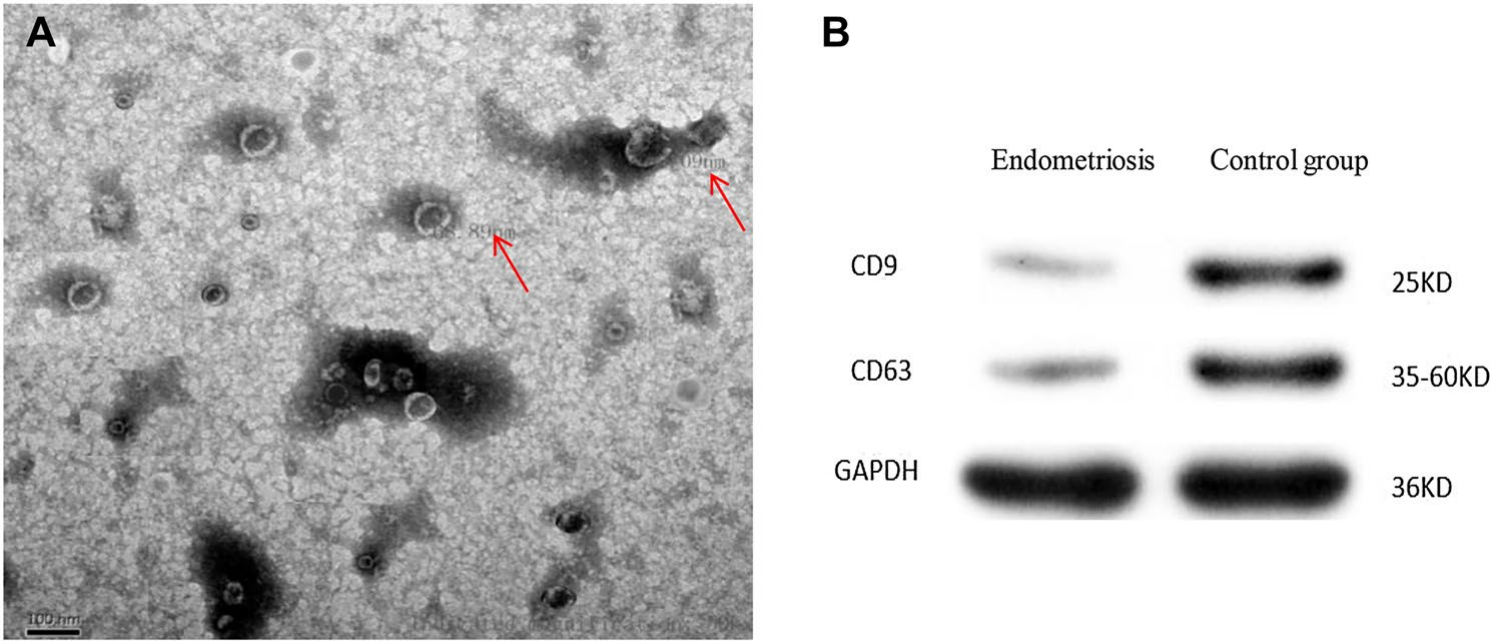
Verification of exosome isolation. **A** Scanning electron microscopy of exosomes in suspension. The red arrows indicate exosomes. Scale bar, 100 µm. **B** Western blot detection of CD9 and CD63 expression by the exosomes

Electron microscopic analysis of exosomes samples showed many disc-like structures with roughly the same shape and size. The edges of the structures were clear and light with concentrated staining in the central area. The particle sizes ranged from about 60–80 nm (Fig. 1A). Expression of both CD9 and CD63, exosome surface marker proteins, was observed on exosomes from both the endometriosis and control groups (Fig. 1B).

### Differential exosomal miRNA expression between the endometriosis and control groups

**Fig. 2:**
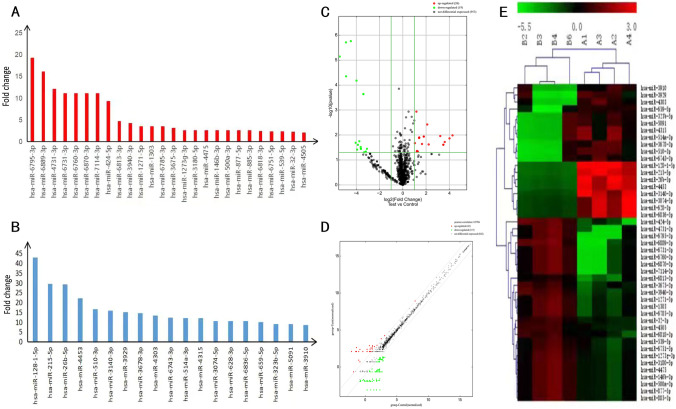
Differential expression of miRNAs between the endometriosis and control groups. **A** Fold change values for 26 up-regulated genes; **B** fold change values for 19 down-regulated genes. **C** Volcano plot of microarray data. **D** Scatter plot analysis of the differential expression of miRNAs in the endometriosis and control groups. **E** Cluster analysis of some differentially expressed miRNAs

Four exosomal samples from each group were submitted for miRNA chip analysis. The four samples from the endometriosis group represented 1 case of stage I endometriosis, 2 cases of stage III endometriosis, and 1 case of stage IV endometriosis. The four samples from the control group represented 1 case of a simple ovarian cyst and 3 cases of ovarian mature teratomas. A total of 45 miRNAs showed significantly differential expressions between the two groups (fold change > 2 and *P* < 0.05). Of these, 26 were up-regulated (including hsa-miR-6795-3p, hsa-miR-146b-3p, hsa-miR-32-3p, hsa-miR-424-5p, and hsa-miR-500a-3p, Fig. 2A) and 19 down-regulated (including hsa-miR-128–1-5p, hsa-miR-215-5p, hsa-miR-26b-5p, hsa-miR-510-3p, and hsa-miR-514a-3p, Fig. 2B) in the endometriosis samples compared with the control samples. The volcano plot also showed a significant difference in the miRNA expression in the two groups (Fig. 2C, differentially expressed miRNAs in red). The results of scatter plot analysis (Fig. 2D) and hierarchical clustering analysis (Fig. 2E) confirmed these findings.

### Prediction of target genes of partially differentially expressed miRNAs

**Fig. 3:**
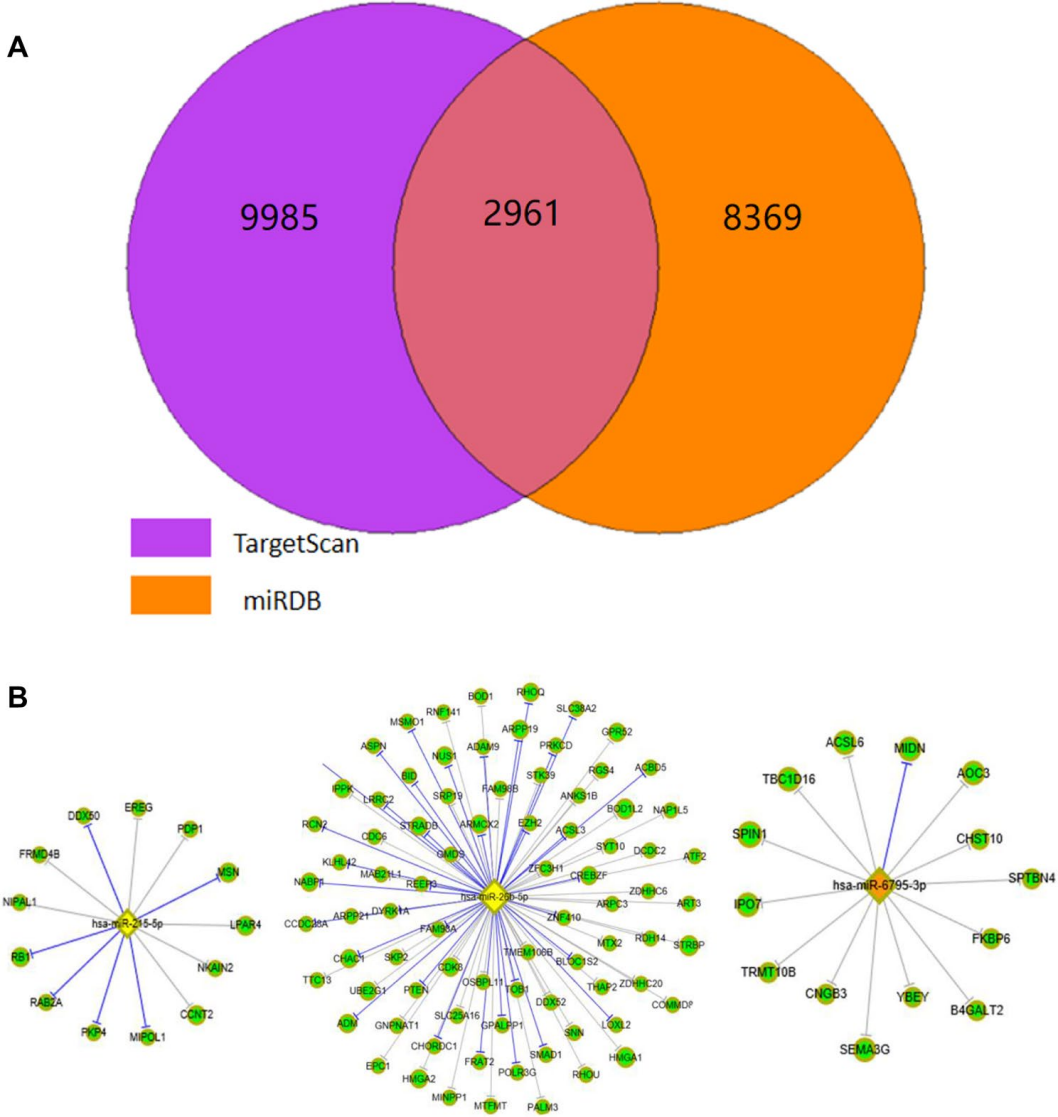
**A** Venn diagram of predicted target gene results from TargetScan 7.2 and miRDB. **B** Partial target genes predicted for miR-26b-5p, miR 215-5p, and miR-6795-3p

Target genes were predicted using online software TargetScan 7.2 and miRDB for significantly differentially expressed miRNAs. A total of 2961 target genes were predicted by both prediction software programs (Fig. 3A, B). These genes (including miR-26b-5p, miR-215-5p, and miR-6795-3p) were included in the subsequent analysis.

### GO and KEGG pathway analyses of target genes of partially differentially expressed miRNAs

**Fig. 4:**
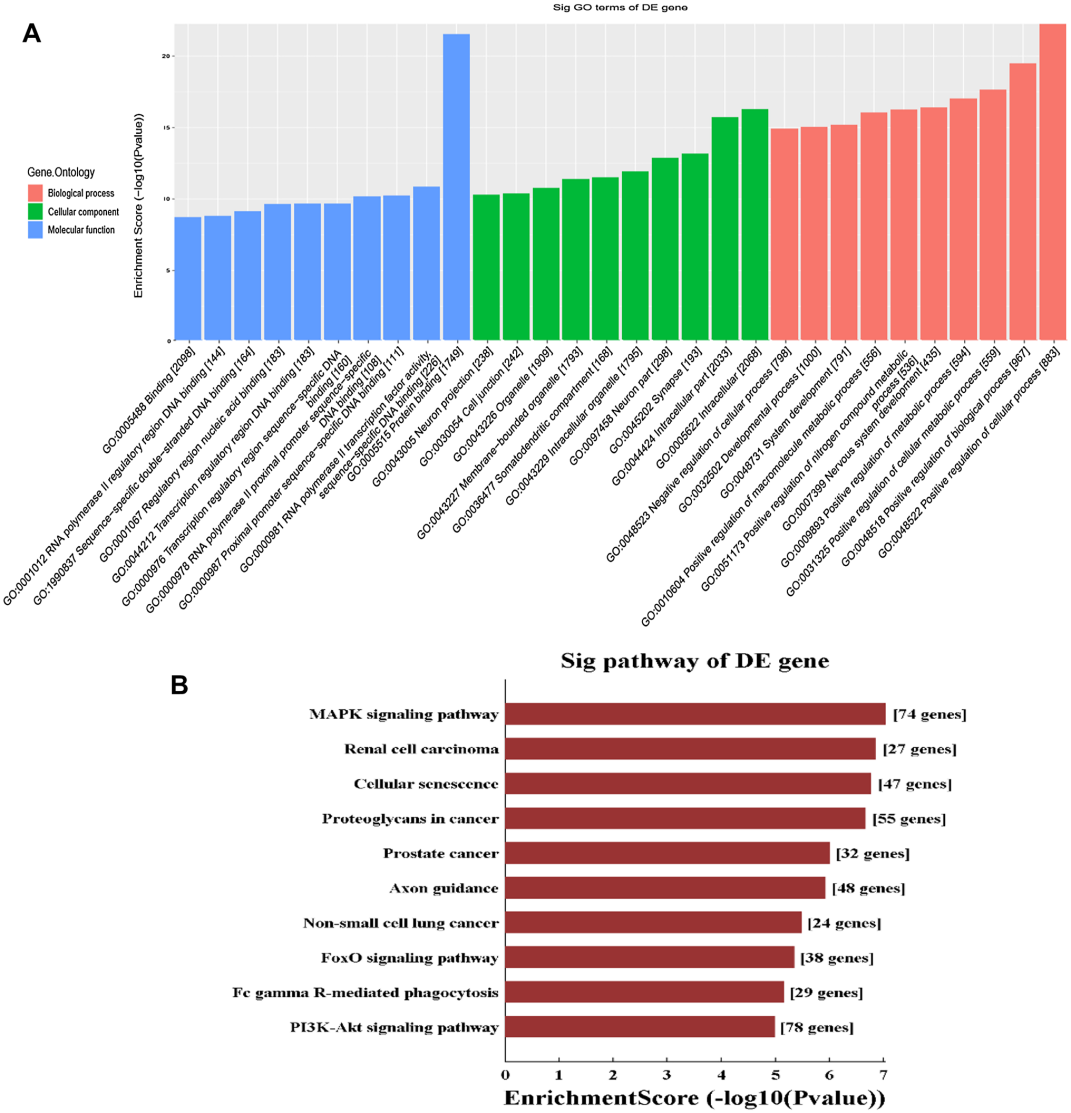
**A** GO analysis and **B** KEGG pathway analysis of target genes of differentially expressed miRNAs

To further explore the potential roles of the significantly differentially expressed miRNAs in the pathogenesis of endometriosis, GO enrichment and KEGG pathway analyses were performed on the significantly differentially expressed miRNAs. GO analysis was mainly carried out according to three aspects: biological process, molecular function, and cell composition. The results showed that the miRNAs were mainly enriched in RNA polymerase II transcription factor activation, protein binding, the composition of synaptic vesicular phosphatase and intracellular structure, and metabolism (Fig. 4A). KEGG pathway analysis showed that the predicted target genes were involved in a variety of signaling pathways, mainly the MAPK and PI3k-AKT signaling pathways (Fig. 4B).

### qRT-PCR verification of partial differential expression of miRNAs

**Fig. 5:**
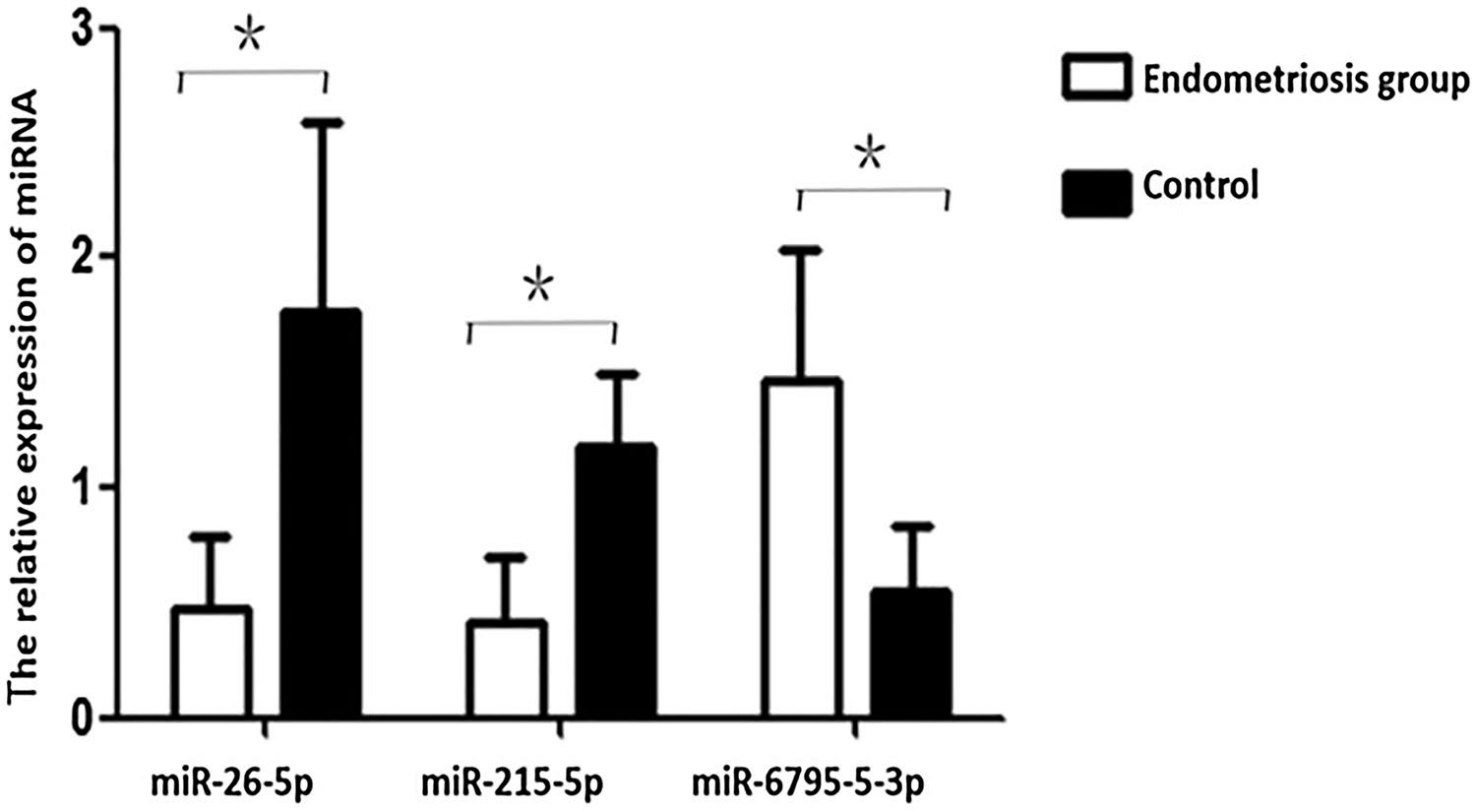
qRT-PCR confirmation of the differential expression of exosomal miRNAs. Data are mean ± standard deviation. *P < 0.05

To confirm the differential expression of the miRNAs identified by the chip analysis, qRT-PCR analysis was performed on serum exosome samples from 42 patients with endometriosis and 24 controls. We found that miR-26b-5p and miR 215-5p expression was down-regulated and miR-6795-3p expression was up-regulated in endometriosis patients compared with controls (all *P* < 0.05), consistent with the results of the miRNA chip analysis (Fig. 5). These results indicate that these genes can serve as potential biomarkers for ovarian endometriosis.

### Value of serum exosomal miRNA in the staging of endometriosis

There were no significant differences in basic characteristics such as age, BMI and menstrual phase between the two groups (Table 1). First, subgroup analyses were performed to compare miRNA expression between cases with stage I–II versus stage III–IV endometriosis; endometriosis with dysmenorrhea versus endometriosis without dysmenorrhea; and endometriosis with infertility versus endometriosis without infertility (Supplemental Figs. S1–3 and Table 2). These results indicate that the serum exosomal miR-26b-5p (Supplemental Fig. S1), miR-215-5p (Supplemental Fig. S2) and miR-6795-3p (Supplemental Fig. S3) can be used not only to distinguish patients with ovarian endometriosis from negative individuals and distinguish between patients with stage I–II and stage III–IV ovarian endometriosis but also to distinguish between endometriosis patients with infertility or dysmenorrhea from those without infertility or dysmenorrhea.

**Table 1:** Characteristics of patients with and without ovarian endometriosis

	Endometriosis group (*n* = 42)	Control group (*n* = 24)	*Z* or *χ*^2^ statistics	*P* value
Age in years, mean ± SD	34.74 ± 7.59	33.54 ± 8.06	− 0.59	0.56
Range of age (years)	21–48	19–48	–	–
BMI, mean ± SD	21.10 ± 2.02	21.53 ± 2.00	− 0.53	0.60
Menstrual cycle (*n*): P/S	16/20	14/11	0.49	0.49
Smoking, *n* (%)	16/42 (38.10)	8/24 (33.33)	0.15	0.70
Alcohol intake, *n* (%)	24/42 (57.14)	13/24 (54.17)	0.06	0.82

*SD* standard deviation, *BMI* body mass index (BMI), *P* proliferative phase, *S* secretory phase

**Table 2:** Relative miRNA expression in exosomes from patients with endometriosis of different stages with or without dysmenorrhea and infertility

miRNA	Stage		Dysmenorrhea		Infertility	
	I–II	III–IV	+	−	+	−
miR-26b-5p	0.51 ± 0.27	0.18 ± 0.14*	0.27 ± 0.13	0.55 ± 0.34*	0.45 ± 0.03	0.51 ± 0.10
miR-215-5p	0.63 ± 0.45	0.31 ± 0.09*	0.66 ± 0.19	0.47 ± 0.35	0.25 ± 0.07	0.66 ± 0.29*
miR-6795-3p	0.99 ± 0.27	1.79 ± 0.32*	1.82 ± 0.29	1.17 ± 0.31*	2.03 ± 0.32	1.16 ± 0.20*

+ indicates that patients with endometriosis have dysmenorrhea or infertility

*A significant difference among the groups (*P* < 0.5)

## Discussion

The results of the present study demonstrated the differential expression of miR-26b-5p, miR 215-5p, and miR-6795-3p in serum exosomes of women with and without endometriosis, and that the expression levels of miR-26b-5p, miR 215-5p, and miR-6795-3p were related to the stage of endometriosis. Additionally, KEGG pathway analysis showed that the predicted target genes were mainly involved in the MAPK and PI3k-AKT signaling pathways. These findings suggest that serum miR-26b-5p, miR-215-5p, and miR-6795-3p expression can be used for evaluating the severity of ovarian endometriosis.

### Analysis of differential exosomal miRNAs promoting endometriosis through signaling pathways

The prediction and functional enrichment analysis of target genes for differentially expressed miRNAs in this study revealed a variety of important signaling pathways that may be involved in the development of endometriosis, including the MAPK signaling pathway and the PI3K-Akt signaling pathway. The MAPK signaling pathway is essential in regulating many cellular processes including inflammation [21], the cell stress response, cell differentiation, cell division, cell proliferation, metabolism, motility, and apoptosis [22, 23]. Additionally, as an estrogen-dependent disease, endometriosis involves activation of the P38/MAPK signaling pathway to promote endometrial cell proliferation and differentiation [22]. The PI3K-AKT signaling pathway also can regulate cell growth, proliferation, differentiation, and apoptosis [23–25]. Additional in vitro experiments showed that blockage of the PI3K pathway inhibits the proliferation of endometriosis epithelial cells and stromal cells [26]. Based on the consistency of these findings with previous studies indicating the roles of these pathways in endometriosis, differentially expressed exosomal miRNAs may participate in the development of endometriosis by regulating the MAPK and PI3K-Akt signaling pathways.

### Analysis of the correlation between serum miR-26b-5p, miR-215-5p, and miR-6795-3p and the severity of endometriosis

In this study, we found that the expression of serum exosomal miR-26b-5p was significantly lower in patients with endometriosis, and its expression was significantly lower in patients with advanced stages versus early stages of endometriosis. This significant down-regulation of miRNA -26b-5p in serum exosomes of patients with endometriosis was identified by both the gene chip analysis and qRT-PCR. The prediction of miRNA -26b-5p target genes by online software revealed that phosphatase and tensin homolog (PTEN) is one of the most important target genes of miRNA-26-5p. PTEN is closely linked to biological behaviors such as cell growth, proliferation, apoptosis, adhesion, and invasion [27]. Xia et al. [28] demonstrated low expression in PTEN in the in situ endometrial tissue of endometriosis patients and found that PTEN expression is closely correlated with the invasiveness of the in situ endometrial gland epithelium. PTEN can act on PI3K/AKT signaling pathways, inhibiting angiogenesis or reducing vascular endothelial growth factor (VEGF) expression [29]. Therefore, it is speculated that the low expression of exosomal miR-26b-5p in the serum of patients with endometriosis may lead to reduced expression of the target gene PTEN, which could weaken the blocking effect on the PI3K/AKT signaling pathway, thus promoting angiogenesis and thereby providing nutrition for the implantation of ectopic endometrium and promoting the survival and growth of ectopic endometrium to participate in the progression of endometriosis.

We also observed that the serum exosomal expression of miR-215-5p was significantly lower in the endometriosis group than in the control group, and its expression in patients with stage III–IV endometriosis than in those with the stage I–II endometriosis, which indicates that miR-215-5p is not only involved in the occurrence of endometriosis but also closely related to the progression of endometriosis. Abnormal expression of miR-215-5p in multiple tumors [30] suggests that it plays an important role in the development of many types of tumors, such as breast cancer [31], brain glioma [32], non-small cell lung cancer [33], colorectal cancer [34], ovarian cancer [35], and cervical cancer [36], and studies have demonstrated its involvement in malignant biological behaviors such as tumor cell proliferation, invasion, migration, anti-apoptosis, and drug resistance [37]. The target gene prediction analysis for miR-215-5p target genes in our study identified CXCL2 as one of the important target genes of miR-215-5p. CXCL2 is a member of the CXC sub-chemokines, is mainly produced by activated macrophages, has a strong chemotactic effect on neutrophils, and is involved in promoting angiogenesis, tumor cell growth, etc. [38, 39]. CXCL2 can not only directly participate in the immune regulation and inflammatory response in vivo, but also can promote neutrophil chemotaxis, smooth muscle migration, angiogenesis, bone remodeling, endothelial cell chemotaxis, and tumor cell growth after specific binding of CXCLR2 to its receptors [40]. Studies have found that CXCL2 pathway activation can lead to the release of inflammatory mediators such as protease, prostaglandin (PG), leukotriene (LT), and reactive oxygen species intermediates, and expression of CXCL2 is high in the peritoneal fluid of patients with endometriosis [41]. The abnormal expression of chemokines may contribute to the development of endometriosis by promoting the inflammatory reaction within the abdominal cavity and changing the microenvironment of the abdominal cavity. We speculate that the silencing effect of miR-215-5p on its target gene CXCL2 is weakened in women with endometriosis, which results in high expression of CXCL2 and supports the progression of endometriosis.

The current research on miR-6795-3p is very sparce. One study has reported that it is differentially expressed in mesenchymal stem cells infected with enterovirus 71 (EV71) [42], but no further experimental analysis was done. In the present study, we found that miR-6795-3p was significantly up-regulated in the serum exosomes of patients with endometriosis by both gene chip and RT-PCR analyses. Our further analysis also showed that its expression level correlated with different stages of endometriosis. However, the mechanism for its role in endometriosis requires additional research.

### Analysis of the relationship between differential exosomal miRNA and endometriosis with infertility

Variation in serum exosomes was related to endometriosis with infertility. Through our further analysis of the experimental data, we found that miR-215-5p expression was lower in endometriosis patients with infertility than in those without infertility, and miR-6795-3p expression was higher. High expression of CXCL2 caused by the weakened silencing effect of miR-215-5p promotes a local inflammatory response, pelvic adhesion, tubal distortion, obstruction, and an abdominal immune microenvironment, which are important factors affecting pregnancy outcomes [41]. The mechanism of miR-6795-3p in infertility requires further study.

### Analysis of the relationship between differential exosomal miRNA and endometriosis with infertility

The differential expression of some serum exosomal contents may give rise to dysmenorrhea. Our study found that miR-26-5p is significantly down-regulated in endometriosis patients with dysmenorrhea, and miR-6795-3p was up-regulated. MiR-26-5p is currently thought to be associated with local inflammation and neuroendocrine regulation, and it can inhibit the expression of estrogen receptor [43]. We speculate that low expression of miR-26b-5p can reduce the inhibitory effect of estrogen receptor expression, promote the release of estrogen-mediated inflammatory signals, and aggravate the local inflammatory effect to cause dysmenorrhea. However, this hypothesis requires further experimental confirmation. Regarding the possibility that miR-6795-3p causes dysmenorrhea, there is no clear mechanism yet.

Some scholars found the differential expression of miR-22-3p and miR-320a in serum exosomes of endometriosis [44]. The incomplete consistency between the research results of other scholars and our experimental conclusions may be caused by the differences in the inclusion criteria for study participants. The patients included in the present study had ovarian endometriosis, and we did not include patients with deep invasive endometriosis.

The following limitations of the study must be addressed in future research. First, a larger-scale cohort miRNA chip analysis will be more convincing. Second, we can further study the regulatory mechanism between these miRNAs and endometriosis at the cellular and molecular levels, especially the pathophysiologic mechanism of miR-6795-3p in endometriosis and related symptoms, which is understudied at present.

## Conclusions

In summary, the present study demonstrated the differential expression of miR-26b-5p, miR-215-5p, and miR-6795-3p in serum exosomes of women with endometriosis compared with women without endometriosis. Additionally, the miR-26b-5p, miR-215-5p, and miR-6795-3p expression levels correlated with the staging of endometriosis. These genes can be used to assess the severity and certain symptoms of ovarian endometriosis. In terms of treatment, studies have confirmed that autosecreted exosomes have the advantage of avoiding autoimmune rejection, as well as good targeting and stability [45]. Therefore, exosomes as drug carriers carrying specific miRNAs will provide new possibilities for the treatment of endometriosis.

## Supplementary Information

Below is the link to the electronic supplementary material.

Supplemental Figure S1. Relative expression of serum exosomal miR-26b-5p in subgroups: (A) endometriosis versus control group; (B) endometriosis I-II versus III-IV group; (C) dysmenorrhea versus without dysmenorrhea group; (D) endometriosis with infertility versus endometriosis without infertility group. Data are mean ± standard deviation. *P < 0.05 (TIF 1091 KB)

Supplemental Figure S2. Relative expression of serum exosomal miR-215-5p in subgroups: (A) endometriosis versus control group; (B) endometriosis I-II versus III-IV group; (C) dysmenorrhea versus without dysmenorrhea group; (D) endometriosis with infertility versus endometriosis without infertility group. Data are mean ± standard deviation. *P < 0.05 (TIF 1393 KB)

Supplemental Figure S3. Relative expression of serum exosomal miR-6795-3p in subgroups: (A) endometriosis versus control group; (B) endometriosis I-II versus III-IV group; (C) dysmenorrhea versus without dysmenorrhea group; (D) endometriosis with infertility versus endometriosis without infertility group. Data are mean ± standard deviation. *P < 0.05 (TIF 1623 KB)

## Data Availability

The datasets generated and analyzed during the current study are available from the corresponding author on reasonable request.
