# Supplementary figures and images for: Serum exosomal miRNA from endometriosis patients correlates with disease severity
Source: Arch Gynecol Obstet. 2021 Sep 20;305(1):117–27. doi: 10.1007/s00404-021-06227-z (PMC8782809; doi:10.1007/s00404-021-06227-z)

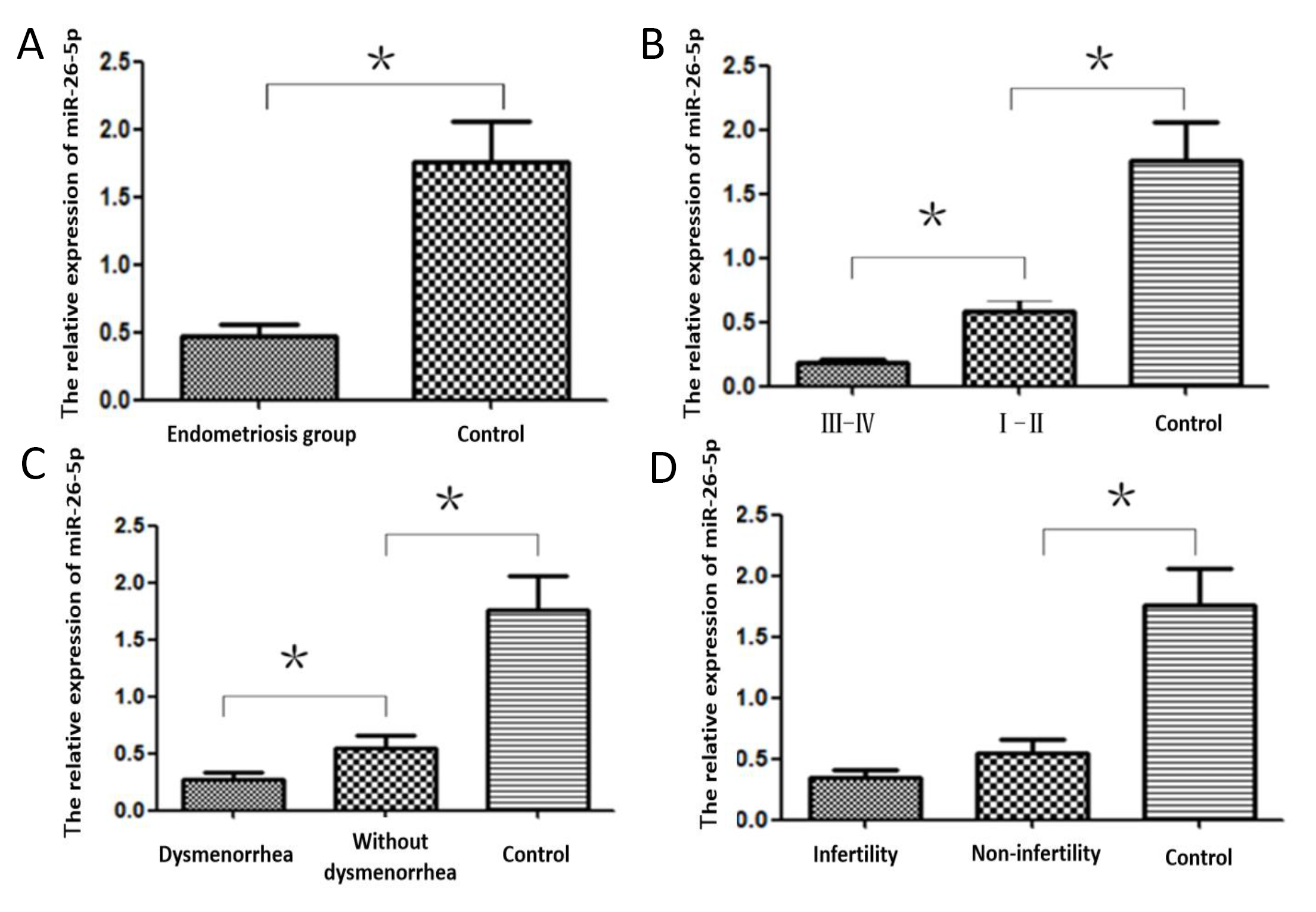

Supplement: Supplementary file 1 — Supplemental Figure S1. Relative expression of serum exosomal miR-26b-5p in subgroups: (A) endometriosis versus control group; (B) endometriosis I-II versus III-IV group; (C) dysmenorrhea versus without dysmenorrhea group; (D) endometriosis with infertility versus endometriosis without infertility group. Data are mean ± standard deviation. *P < 0.05 (TIF 1091 KB) [file 404_2021_6227_MOESM1_ESM.tif]

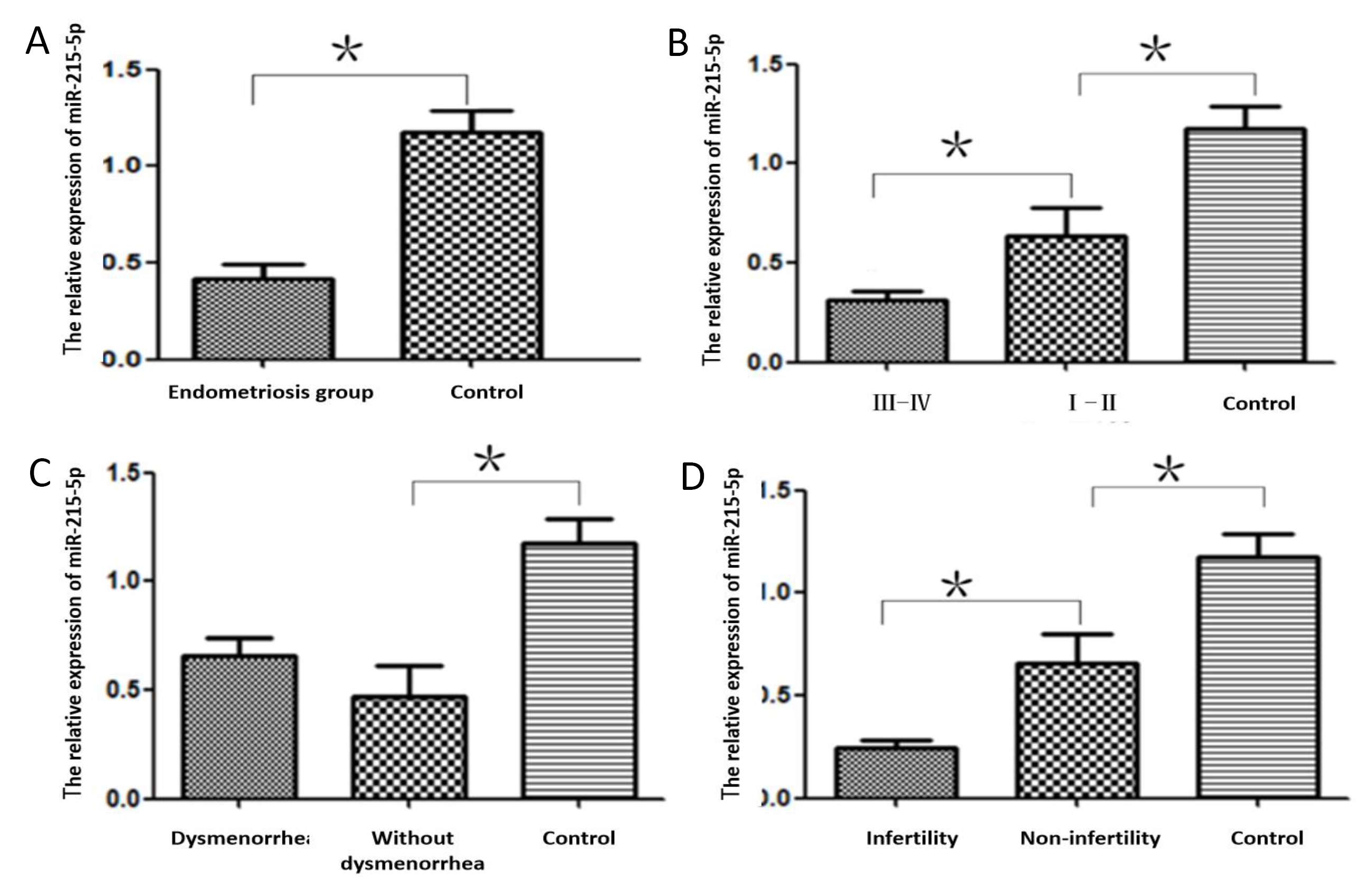

Supplement: Supplementary file 2 — Supplemental Figure S2. Relative expression of serum exosomal miR-215-5p in subgroups: (A) endometriosis versus control group; (B) endometriosis I-II versus III-IV group; (C) dysmenorrhea versus without dysmenorrhea group; (D) endometriosis with infertility versus endometriosis without infertility group. Data are mean ± standard deviation. *P < 0.05 (TIF 1393 KB) [file 404_2021_6227_MOESM2_ESM.tif]

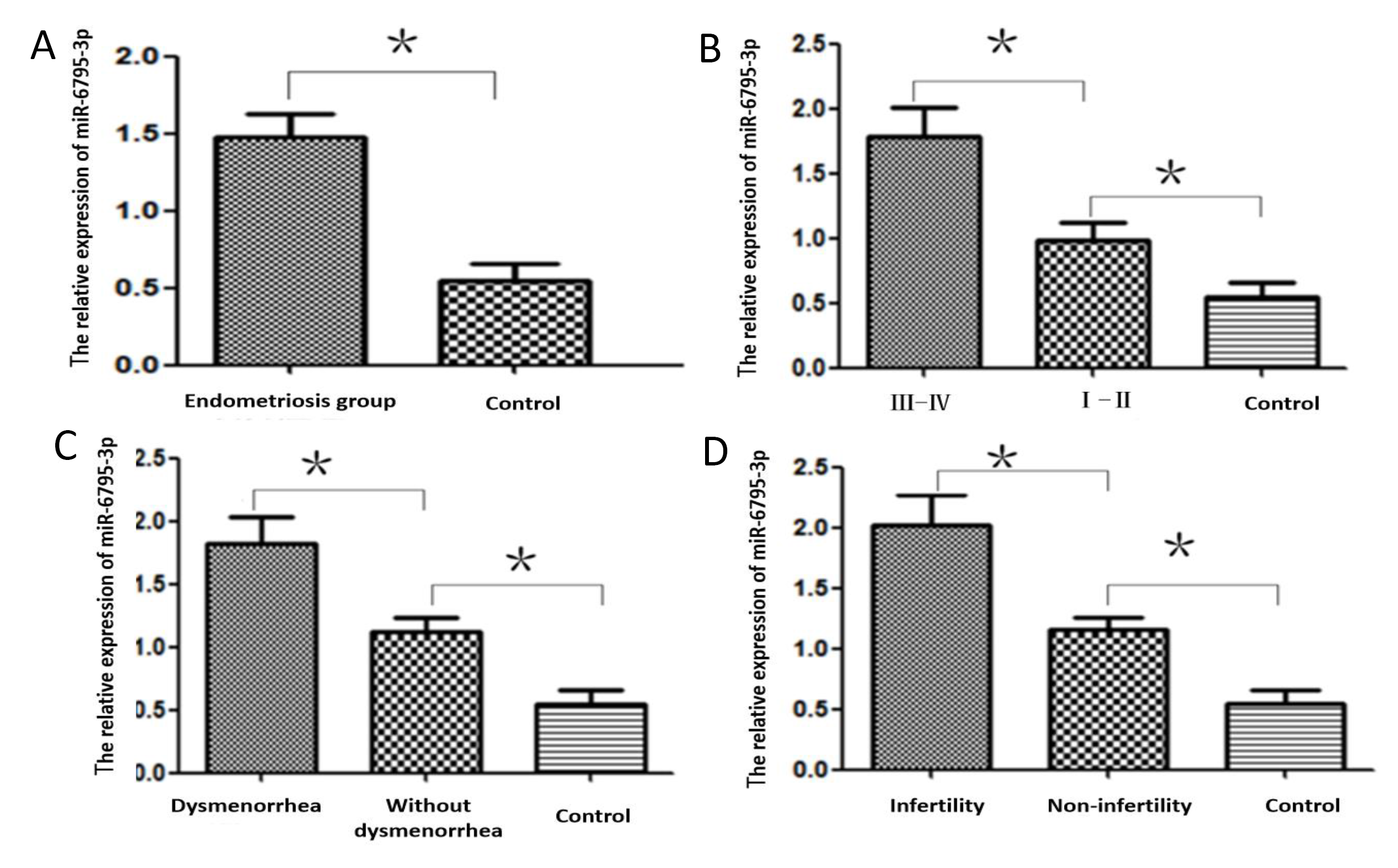

Supplement: Supplementary file 3 — Supplemental Figure S3. Relative expression of serum exosomal miR-6795-3p in subgroups: (A) endometriosis versus control group; (B) endometriosis I-II versus III-IV group; (C) dysmenorrhea versus without dysmenorrhea group; (D) endometriosis with infertility versus endometriosis without infertility group. Data are mean ± standard deviation. *P < 0.05 (TIF 1623 KB) [file 404_2021_6227_MOESM3_ESM.tif]
